# Effects of dietary *Elaeagnus angustifolia* and ginger root powders on performance, egg quality, serum traits, ileal histomorphology, and organ traits of late-phase Lohmann LSL-Lite laying hens

**DOI:** 10.1016/j.psj.2026.107781

**Published:** 2026-09-15

**Authors:** Zahra Hamzehee, Mehran Torki, Khodabakhsh Rashidi, Hamid Pajavand

**Affiliations:** a Department of Animal Science, College of Agriculture and Natural Resources, Razi University, Kermanshah, 67156-85423, Iran; b Research Center of Oils and Fats, Kermanshah University of Medical Sciences, Kermanshah, Iran; c Department of Microbiology, Kermanshah University of Medical Sciences, Kermanshah, Iran

**Keywords:** *Elaeagnus angustifolia powder*, Ginger root powder, Phytobiotic, Late stage of production

## Abstract

The current study evaluated the effects of *Elaeagnus angustifolia* powder (EAP), Ginger root powder (GRP), and their interaction on performance, egg quality, ileum histomorphology, serum metabolites, differential leukocyte counts, and organ characteristics of Lohmann LSL-Lite laying hens. 144 Lohmann laying hens, 92 weeks of age, were assigned to a completely randomized design with four treatments, each comprising six replicates of six birds. For eight weeks, hens received one of four diets: a control diet (without additive), a diet supplemented with 7.5 g/kg EAP, a diet supplemented with 5 g/kg GRP, and a diet supplemented with both 7.5 g/kg EAP and 5 g/kg GRP. A significant interaction between GRP and EAP was observed on egg production and FCR in the second phase (97–100 wk), yolk percentage, pancreas weight, villus height, width and surface area (*P* < 0.05). During weeks 97 to 100 of age, hens fed the experimental diets showed higher egg production, along with a lower FCR, than hens fed the CON (*P* < 0.05). Specific gravity, pancreas weight increased when EAP was used compared to when it was not used, however, yolk percentage and serum TG decreased (*P* < 0.05). Single and combined use of GRP and EAP increased villus height, width, surface area, and SOD, while decreasing MDA (*P* < 0.05). Overall, the findings demonstrate that dietary supplementation with EAP and GRP can improve productive performance, gut health, antioxidant capacity, and metabolic efficiency in aging laying hens.

## Introduction

The period of egg production after 60 weeks of age is generally considered the late phase of laying, during which production performance and egg quality gradually decline (Nik et al., 2025). Advancing age is associated with increased generation of oxidants from different sources and a concurrent reduction in antioxidant enzyme activity, which weakens the primary defense system against oxidative damage (Celano et al., 2012; Pierzchała-Koziec et al., 2024).

Aging triggers an unavoidable physiological decline, largely driven by mitochondrial dysfunction and chronic metabolic stress, which shifts the redox balance toward excessive reactive oxygen species (ROS) production (Lee et al., 2004). In late-phase laying hens, the failure of endogenous antioxidant systems—specifically the attenuated activities of superoxide dismutase and glutathione peroxidase—exacerbates this oxidative stress (Hamzehee et al., 2025; Tong et al., 2015). Consequently, the resulting oxidative damage impairs intestinal morphology and reproductive hormone function, serving as a primary bottleneck for maintaining optimal production performance and egg quality (Park and Sohn, 2018; Yu et al., 2023). According to the findings of Sun et al. (2024), the intestines of laying hens in the late stages of laying usually show lipid metabolism disorders. These disorders are observed simultaneously with a decrease in energy production and antioxidant capacity. In poultry reproduction, the age-related decline of ovarian function is associated with mitochondrial dysfunction, compounded by diminished antioxidant capacity, which ultimately culminates in follicular atresia and reduced egg production (Zhan et al., 2024). Because eggshell quality is essential for protecting internal egg contents and preventing economic losses caused by cracked and broken eggs, maintaining shell integrity is a major concern in commercial layer production (Park and Sohn, 2018). Aging hens experience reduced egg production performance and quality due to cumulative oxidative stress and physiological decline (Lee et al., 2025).

In recent years, phytobiotics have gained attention as natural feed additives capable of improving animal health and productivity while reducing oxidative stress. Phytobiotics are plant-derived compounds that include both primary and secondary metabolites and can beneficially affect growth, metabolism, and health in poultry and other livestock species (Clavijo and Flórez, 2018). Primary metabolites include nutrients such as proteins, fats, and carbohydrates, whereas secondary metabolites comprise essential oils, pigments, bitter compounds, and phenolic substances (Grashorn, 2010). Based on their origin and processing, phytobiotics are commonly classified as herbs, botanicals, essential oils, and oleoresins (Clavijo and Flórez, 2018). Among the various plant-based additives used in poultry nutrition, *Elaeagnus angustifolia* powder (EAP) and Ginger root powder (GRP) have attracted increasing interest because of their bioactive compounds and potential physiological benefits.

Ginger (*Zingiber officinale*) is an important spice and medicinal plant widely cultivated in Asia and used traditionally for its therapeutic properties (Derese et al., 2026; Shahrajabian et al., 2019). Ginger root contains a wide range of biologically active compounds with antioxidant, antimicrobial, and anti-inflammatory effects (Ali et al., 2008). It is considered a promising herbal supplement for improving poultry health and productivity because it may reduce pathogenic intestinal bacteria, enhance digestibility, and improve nutrient absorption (Li et al., 2025; Umatiya et al., 2018). Its major bioactive constituents include gingerols, gingerdiones, and shogaols, with gingerol being the most abundant compound and contributing substantially to its antioxidant activity (Ko et al., 2019; Mahomoodally et al., 2021). Reports indicate that the 6-gingerol and 6-shogaol content in GRP is approximately 10.38 g/kg and 1.34 g/kg, respectively (Zhao et al., 2011). Recent poultry studies have shown that GRP can enhance productivity and physiological health in laying hens (Derese et al., 2025) . A meta-analysis has shown that ginger can improve health indicators, daily egg production, egg mass, and egg weight in laying hens (Ogbuewu and Mbajiorgu, 2022).

*Elaeagnus angustifolia*, commonly known as Russian olive or oleaster, is native to the Irano-Turanian region and contains valuable nutrients and phytochemicals such as vitamins, flavonoids, polyphenols, free fatty acids, and mono- and polysaccharides (Bendaikha et al., 2014; Hoseini et al., 2021). *Elaeagnus angustifolia* exhibits tolerance to challenging soil conditions, including drought, high salinity, and alkalinity, rendering it valuable for ecosystem maintenance in arid environments (Asadiar et al., 2013; Wang et al., 2006). Various components of *E. angustifolia L*., such as roots, wood, bark, flowers, leaves, and fruits, possess medicinal attributes and find applications across the food, health, pharmaceutical, perfumery, and wood industries (Kiseleva and Chindyaeva, 2011; Saboonchian et al., 2014). Although research on EAP in poultry nutrition is still limited, available studies suggest that it may have beneficial effects in poultry nutrition. EAP is abundant in bioactive phenolic compounds, including p-hydroxybenzoic, protocatechuic, 4-hydroxycinnamic, benzoic, ferulic, and vanillic acids (Azmat et al., 2025; Carradori et al., 2020; Hamidpour et al., 2019). It further comprises various flavonoids, such as rutin, quercetin, and luteolin, which contribute to its potent antioxidant capacity (Hamidpour et al., 2017). Moreover, the fruit provides sterols, carotenoids, vitamins, minerals, and fatty acids, along with substantial concentrations of 4-hydroxybenzoic (45.8 mg/100 g dry wt.) and caffeic acids (32 mg/100 g dry wt.), underscoring its nutritional, phytochemical, and biological significance (Ayaz and Bertoft, 2001; Azmat et al., 2025; Bucur et al., 2008). In EAP, total flavonoids and polycarboxylic acids have been quantified at 1.35%, further substantiating its phytochemical richness (Tehranizadeh et al., 2016). For example, dietary inclusion of EAP improved albumen height, Haugh unit, and yolk color in laying hens (Davariyan et al., 2018). In broilers under heat stress, supplementation with EAP and *Zizyphus jujube* improved glutathione peroxidase activity, lowered blood glucose, and enhanced some meat quality traits, even though growth performance was not affected (Basiriyan et al., 2021). These findings indicate that EAP may exert physiological effects relevant to both oxidative status and production efficiency. The inclusion levels of 7.5 g/kg EAP and 5 g/kg GRP were selected based on previous dose–response studies in poultry showing that 0.75% EAP improves antioxidant status and blood metabolites in broilers (Basiriyan et al., 2021), and 0.5% GRP enhances egg production, immune response, and antioxidative status in laying hens and broilers (Akbarian et al., 2011; Al-Khalaifah et al., 2022; Zhang et al., 2009). These doses therefore represent biologically effective and safe levels that are expected to exert complementary antioxidant and metabolic effects when combined.

Given the antioxidant and biological properties of GRP and EAP, it was hypothesized that combining these phytobiotics may produce complementary or synergistic benefits in laying hens. Therefore, the objective of the present study was to evaluate the individual and combined effects of GRP and EAP on production performance, egg quality, blood biochemical and antioxidant parameters, differential leukocyte counts, ileal histomorphology, and organ characteristics in late-phase laying hens. This information may help identify natural nutritional strategies to maintain productive efficiency, and produce safe, high-quality eggs.

## Materials and methods

### Diets and hens

The study was conducted at the Faculty of Agriculture, Razi University, Kermanshah, Iran. All experimental procedures were performed in accordance with institutional and national guidelines for the care and use of laboratory animals in Iran. The study protocol was approved by the Research Ethics Committees of Laboratory Animals at Razi University (Approval ID: IR.RAZI.AEC.1405.007).

A completely randomized design was employed, utilizing 144 Lohmann LSL-Lite laying hens (92 wk of age) allocated to 4 dietary treatments, with 6 replicate cages per treatment and 6 hens per cage (cage serving as the experimental unit), over an 8-week period. For all production performance variables, values were first averaged across the 6 hens within each cage to obtain a single cage-level observation, and this cage-level average was used as the unit of statistical analysis (*n* = 6 cages/treatment). The experiment investigated the impact of supplementing diets with EAP, GRP, and a combination of EAP and GRP. EAP and GRP were both purchased by the Giahine brand (Isfahan, Iran), an established commercial supplier of herbal and medicinal plant powders. EAP consisted of dried fruit powder of *E. angustifolia*, supplied as milled whole fruit. GRP consisted of dried rhizome powder of *Zingiber officinale Roscoe*. EAP and GRP were added to the basal diet at a rate of 7.5 and 5.0 g/kg, respectively. The experimental diets were then stored in sealed containers until feeding. A 2 × 2 factorial arrangement was used, consisting of a basal corn-soybean meal diet with two levels of EAP (0 and 7.5 g/kg diet) and two levels of GRP (0 and 5 g/kg diet). Hence, four dietary treatments were administered: (1) basal diet (control, CON); (2) basal diet + 7.5 g/kg EAP; (3) basal diet + 5 g/kg GRP; and (4) basal diet + 7.5 g/kg EAP + 5 g/kg GRP phytobiotics. No antimicrobial growth promoter was included in the basal diet formulation. The proximate composition of both GRP and EAP was determined using the AOAC (2000) method. The GRP contained 16.20% moisture, 9.16% crude protein, 9.94% crude fiber, 8.38% ether extract, 6.63% ash, and 49.69% nitrogen-free extract, whereas the EAP contained 6.17% moisture, 7.87% crude protein, 75.60% nitrogen-free extract, 6.64% crude fiber, 1.72% ether extract, and 1.71% ash. In addition, according to the manufacturer’s brochure, GRP contained 6-gingerol (3.8 ± 0.19 mg/kg) and 6-shogaol (0.99 ± 0.09 mg/kg), whereas EAP contained total flavonoids and polycarboxylic acids (1.22% ± 0.11). The composition and calculated nutrient content of the basal diet are presented in Table 1.

**Table 1:** Ingredient composition and calculated nutrient content of the basal diets of laying hens.

Ingredients (%)	Amount	Calculate	Amount
Corn	57.94	ME (Kcal/kg)	2711
Soybean meal	24.2	CP (%)	15.38
Oyster	6.10	Lys (%)	0.71
Calcium carbonate	3.64	Met + Cys (%)	0.65
Barley	2.10	Thr (%)	0.51
Rice bran	1.82	Met (%)	0.42
Oil	1.65	Calcium	4.02
DCP	1.20	AP (%)	0.31
Vit & min Premix	0.61	Sodium	0.15
NaCl	0.21		
DL-Methionine	0.21		
bicarbonate sodium	0.17		
toxin Binder	0.08		
Phytase	0.04		
Acidifier	0.03		

Note: Premix composition: Each 10 kg contained vitamin D3 (2,000,000 IU), vitamin A (12,000,000 IU), vitamin E (8,000 IU), vitamin K (2,000 mg), vitamin B1 (2,000 mg), vitamin B2 (500 mg), vitamin B6 and vitamin B12 (12,000 µg), vitamin C (25,000 mg), calcium-d-pantothenate (6,000 mg), niacin (40,000 mg), choline chloride (10,000 mg), lysine (30,000 mg), manganese (120,000 mg), iron (20,000 mg), iodine (200 mg), zinc (100,000 mg), cobalt (200 mg), copper (4,000 mg), and zinc bacitracin (21,000 mg).

### Management and production traits

The ambient temperature within the poultry house was maintained within a range of 18°C to 20°C. Hens were housed in physical cages measuring 44 × 40 × 40 cm, with 3 birds per physical cage. Two adjacent physical cages sharing a common wall were combined to form a single experimental unit (replicate) of 6 hens, resulting in 6 replicates per treatment. A trough feeder and a nipple drinker were used. Mechanical ventilation was provided throughout the experimental facility. Light intensity and relative humidity within the experimental facility were maintained at approximately 15 lux and 60%, respectively. Before commencing the experiment, a seven-day monitoring period was conducted to assess body weight, production performance, egg weight, and egg quality, thereby ensuring an equitable distribution of replicates. Analysis of variance confirmed no significant differences among treatments. A one-week adaptation period was implemented before the start of the experiment. Birds were housed in cages for eight weeks under a 16 h light and 8 h dark photoperiod. FI was recorded at 7-day intervals, expressed as g/hen/day, and calculated based on feed weights measured at the beginning and end of each period. Egg production percentage and egg mass (g/hen/day) were calculated as (1) and (2):

(1) Egg production (%) = (total number of eggs produced / total hen-days) × 100

(2) Egg mass = egg production (%) × average egg weight (g) / 100

FCR was determined as kg feed/kg egg (total FI divided by total egg mass). Total eggs were collected daily and weighed individually to ascertain egg weight. Mortality was recorded as it occurred, and the body weight of dead birds was measured. Egg production and FI were corrected for mortality. Mortality rates during the 8-week experimental period were 8.33, 5.56, 2.78, and 2.78% for CON, EAP, GRP, and MIX treatments, respectively. No statistically significant differences were observed among treatments (*P* > 0.05). Performance parameters were computed based on egg production, egg mass, and egg weight.

### Egg quality characteristics

Egg quality traits were evaluated at the end of the experiment. Four eggs were randomly collected from each replicate (cage) to assess both external and internal quality traits; the values of the four eggs were pooled and averaged to obtain a single replicate-level mean, which was used as the experimental-unit value for statistical analysis (*n* = 6 replicates per treatment).

Individual egg weights were meticulously documented, and shape index was subsequently calculated utilizing the formula (3):

(3) Shape index = (egg width ÷ egg length) × 100

Egg dimensions (width and length) were quantified with a digital caliper. Specific gravity was determined according to the method described by Butcher and Miles (1991).

Shell thickness was measured at three points (air cell, equator, and sharp end) using a micrometer, after removal of the shell membrane, and the average of the three measurements was recorded. Shell strength was measured using a digital egg shell strength tester (EG-001, Imada Inc., Northbrook, IL, USA), which applies a compressive force to the egg and automatically records the peak force (N) required to fracture the shell.

Each egg was then broken onto a flat surface, and albumen height and yolk height were measured using a tripod micrometer. yolk index was calculated as yolk height divided by the average yolk diameter. Yolk color was assessed using the Roche Yolk Color Fan. After separating the yolk from the albumen, yolk weight was determined using a digital scale with 0.01 g precision. Shell weight was determined after air-drying the shell, and albumen weight was calculated as egg weight minus the sum of yolk weight and shell weight. Haugh units were determined using the subsequent formula (4):

(4) $$\text{Haugh}\;\text{units}:\;100\;\times \;\text{log}\;\left(H+7.6\;-\;1.7W^{0.37}\right)$$

where H represents the albumen height (mm) and W denotes the egg weight (g).

### Blood biochemical parameters and anti-oxidant status

Blood samples were collected from the brachial vein of one bird per replicate. For optimal serum separation, blood samples were incubated for 2 hours at 37°C and then centrifuged at 5000 rpm for 15 minutes at 4°C. The resulting serum was collected and sent to Jam Laboratory (Kermanshah, Iran) for all analyses. Serum concentrations of glucose (GLU), triglycerides (TG), total cholesterol (TC), total protein (TP), albumin (ALB), calcium (Ca), phosphorus (P), alanine aminotransferase (ALT), aspartate aminotransferase (AST), and alkaline phosphatase (ALP) were determined using AUDIT kits (Isfahan, Iran) according to the company's instructions and using a Beckman coulter AU 408 analyzer (USA). Serum concentrations of thiobarbituric acid-reactive substances (TBARS), expressed as malondialdehyde (MDA) equivalents, were determined using the Nalondi™ Lipid Peroxidation Assay Kit (version 0.84; Navand Salamat Co., Urmia, Iran), based on the reaction of TBARS with thiobarbituric acid (TBA) at elevated temperature to form a colored MDA-TBA complex. glutathione peroxidase (GSH-Px) activity was determined using the Nagpix™ Glutathione Peroxidase Assay Kit (version 0.9; Navand Salamat Co., Urmia, Iran), superoxide dismutase (SOD) activity was determined using the Nasdox™ Superoxide Dismutase Assay Kit (version 0.6; Navand Salamat Co., Urmia, Iran), and serum total antioxidant capacity (TAC) was determined using the Naxifer™ Total Antioxidant Capacity Assay Kit (version 0.8; Navand Salamat Co., Urmia, Iran).

### Differential leukocyte counts

At the end of the period (end of 100 weeks), one bird per replicate was randomly selected from each treatment, blood was drawn from the brachial vein and then a blood smear was prepared. Then, it was fixed with absolute ethyl alcohol. Then, the fixed samples were stained using Giemsa stain and white cells were counted using a microscope and a 100 lens and the number of each group was recorded as a percentage and the samples and cell types were photographed using a digital camera and different filters.

### Ileum histomorphology characteristics

Hens were slaughtered following standard poultry slaughter protocols. For histomorphology evaluation, one 2 cm segment of the distal ileum was collected from one hen per replicate at the end of the trial (*n* = 6 hens/treatment), subsequently rinsed with distilled water, and then transferred to a sample container containing 10% formaldehyde. The tissue samples were processed using a Dided Sabz Co. D2080/H tissue processor (Iran), followed by paraffin embedding and sectioning with a Leica rotary microtome. Eight serial sections of 4 µm thickness were prepared. Sections were examined using an Olympus BH2 light microscope (Olympus, Tokyo, Japan) equipped with 10 × and 40 × objective lenses, and digital images were captured and analyzed using Dino Capture image-analysis software (Dino-Lite, Taiwan). For each bird, eight well-oriented, intact villus-crypt units were selected, and the mean value of the measured parameters was calculated for each bird; only vertically oriented villi with clearly defined tips and crypt bases were included to ensure consistency and minimize measurement bias. All slides were labeled with numerical codes rather than treatment identifiers, and all morphometric evaluations were performed blinded to treatment by the Research Center of Oils and Fats, Kermanshah University of Medical Sciences (Kermanshah, Iran). Histological evaluation was performed on sections stained with hematoxylin and eosin. Morphological measurements of intestinal villi, encompassing villus height, villus width, crypt depth, and the villus height to crypt depth ratio, were precisely determined via microscopy.

Villus surface area (VSA) was calculated for all groups, regardless of villus shape, using a formula described by Sakamoto et al. (2000). VSA was quantified using formula (5):

(5) VSA=2π×(VW/2)×VH

where π = 3.14, VW represents villus width (µm), and VH denotes villus height (µm).

### Organ characteristics

At the end of the experimental period (100 wk of age), hens were fasted for 12 h and then one bird per replicate (six birds per treatment) was slaughtered by the cervical dislocation. Live body weight was recorded immediately before slaughter. After slaughter, feathers, head, feet, and viscera were removed, and carcass weight was recorded and expressed as a percentage of live body weight (carcass weight / live body weight × 100). The weights of the gizzard, pancreas, liver, and heart were recorded using a digital scale with 0.1 g precision and expressed as a percentage of live body weight (organ weight / live body weight × 100). After removal of intestinal contents, the weights of the duodenum, jejunum, ileum, and cecum were recorded using a digital scale with 0.1 g precision and expressed as a percentage of total intestinal weight (segment weight / total intestinal weight × 100).

### Data analysis

The experiment employed a 2 × 2 factorial arrangement, with EAP (0 or 7.5 g/kg) and GRP (0 or 5 g/kg) as fixed effects. The cage served as the experimental unit for production performance variables (*n* = 6 replicates/treatment), and the averaged replicate-level value of 4 eggs per cage served as the experimental unit for egg quality traits (*n* = 6 replicates/treatment). For blood biochemical, antioxidant, differential leukocyte counts, ileal histomorphology, and organ variables, one hen was randomly selected from each replicate cage for sampling, and the value obtained from this single bird served as the experimental unit (*n* = 6 replicates/treatment). Because production performance data were first summarized into period-level (92–96, 97–100, and 92–100 wk) cage means prior to analysis, a repeated-measures/mixed model incorporating time as a within-subject factor was not applied; each period was instead analyzed independently using the General Linear Model (GLM) procedure. Normality of data distribution was assessed using the Shapiro-Wilk test, and homogeneity of variance was assessed. Data were analyzed using the GLM procedure in SAS software (version 9.4). Tukey's honest significant difference (HSD) test was applied for mean comparisons, with significance declared at *P* < 0.05. The following statistical model (4) was used for the 2 × 2 factorial analysis of variance to test the main effects of EAP, GRP, and their interaction:

(6) $$Y_{ijk}=\mu +a_{i}+b_{j}+{\left(ab\right)}_{ij}+e_{ijk}$$

Where $Y_{ijk}$ is the observed value, μ is the overall mean, $a_{i}$ is the effect of EAP, $b_{j}$ is the effect of GRP, ${\left(ab\right)}_{ij}$ is the interaction effect between factors, and $e_{ijk}$ is the experimental error. To facilitate direct comparison among the four treatment combinations (CON, EAP, GRP, and MIX), treatment means were additionally compared using the following one-way model (5):

(7) $$Y_{ij}=\mu +t_{j}+e_{ij}$$

This treatment-level model was primarily used when a significant EAP × GRP interaction was detected, to allow multiple-comparison testing among the four individual treatment combinations.

## Results

### Production performance

Table 2 details the impact of various dietary treatments on the production performance of Lohmann LSL-lite laying hens, specifically examining egg production, egg weight, egg mass, FI, and FCR. Dietary supplementation with EAP, GRP, and their combination (MIX) led to increased egg production during the second period (97–100 wk) compared to the CON (*P* < 0.05). Furthermore, GRP supplementation exhibited a significant improvement over the entire experimental period (92–100 wk; −6.57%; *P* = 0.046) when compared to the non-GRP group. During the second period, all supplemented groups (EAP, GRP, and MIX) exhibited a lower FCR than the CON (*P* = 0.047).

**Table 2:** Effect of dietary *Elaeagnus angustifolia* powder and ginger root powder on production performance of Lohmann laying hens during 92–96, 97–100, and 92–100 weeks of age.

Trait	Treatment means				SEM	Main-effect means				SEM	*P*-Values		
						EAP (g/kg of diet)		GRP (g/kg of diet)					
	CON	EAP	GRP	MIX		–	7.5	–	5		EAP	GRP	EAP × GRP
Egg Production (%)													
92–96 wk	77.52	81.32	85.36	83.11	2.857	81.44	82.22	79.42	84.23	2.021	0.789	0.108	0.303
97–100 wk	75.70^b^	87.40^a^	83.57^ab^	81.31^ab^	3.258	79.63	84.35	81.54	82.44	2.303	0.163	0.789	0.045
92–100 wk	76.61	84.36	84.46	82.21	2.639	80.54	83.28	80.49	83.33	1.867	0.311	0.294	0.073
Egg Weight (g/egg)													
92–96 wk	66.21	66.19	66.15	66.66	0.984	66.18	66.43	66.20	66.41	0.696	0.804	0.834	0.789
97–100 wk	64.80	64.55	64.34	65.62	0.995	64.57	65.08	64.68	64.98	0.704	0.612	0.766	0.453
92–100 wk	65.50	65.37	65.25	66.14	0.940	65.38	65.76	65.44	65.69	0.665	0.689	0.788	0.592
Egg Mass(g/d)													
92–96 wk	51.33	53.81	56.36	55.39	1.869	53.84	54.60	52.57	55.57	1.322	0.689	0.092	0.368
97–100 wk	49.02	56.39	53.73	53.42	2.227	51.37	54.91	52.70	53.58	1.575	0.129	0.699	0.099
92–100 wk	50.19	55.13	55.02	54.40	1.786	52.61	54.76	52.66	54.71	1.263	0.241	0.264	0.136
Feed Intake (g/d/bird)													
92–96 wk	115.55	115.32	114.81	113.77	1.226	115.18	114.55	115.43	114.29	0.867	0.612	0.361	0.741
97–100 wk	107.71	108.14	102.49	104.56	2.317	105.100	106.35	107.93	103.53	1.638	0.595	0.072	0.726
92–100 wk	111.63	111.73	108.65	109.16	1.572	110.14	110.45	111.68	108.91	1.112	0.847	0.093	0.898
Feed Conversion Ratio (kg/kg)													
92–96 wk	2.26	2.18	2.04	2.07	0.080	2.15	2.12	2.22	2.05	0.056	0.742	0.055	0.526
97–100 wk	2.21^a^	1.93^b^	1.92^b^	1.97^b^	0.077	2.07	1.95	2.07	1.95	0.055	0.150	0.139	0.047
92–100 wk	2.23	2.05	1.98	2.02	0.065	2.10	2.03	2.13^a^	1.99^b^	0.046	0.284	0.046	0.102

CON = Control diet (without additive); EAP: *Elaeagnus angustifolia* powder; GRP: Ginger root powder; MIX = basal diet + *Elaeagnus angustifolia* powder and Ginger root powder. experimental units: (*n* = 6 cages/treatment). Means within a row with different superscripts differ significantly (*P* < 0.05).

### Egg quality parameters

Table 3 presents the effect of dietary treatments on egg quality. A significant EAP × GRP interaction was observed for yolk percentage (*P* = 0.0026); hens fed the combination of EAP and GRP (MIX) had a lower yolk percentage than the other treatment groups. No significant interaction was observed for any other egg quality parameter (*P* > 0.05). EAP supplementation significantly increased specific gravity (*P* = 0.015) but significantly decreased Haugh unit (*P* = 0.043), indicating a reduction in albumen quality. Additionally, GRP supplementation significantly reduced yolk percentage (*P* = 0.015).

**Table 3:** Effect of dietary *Elaeagnus angustifolia* powder and ginger root powder on egg quality parameters of Lohmann laying hens at 100 weeks of age.

Trait	Treatment means				SEM	Main-effect means				SEM	*P*-Values		
						EAP (g/kg of diet)		GRP (g/kg of diet)					
	CON	EAP	GRP	MIX		–	7.5	–	5		EAP	GRP	EAP × GRP
Shape index	78.13	76.51	77.26	76.85	0.536	77.70^a^	76.68^b^	77.36	77.06	0.379	0.062	0.633	0.263
Yolk height(mm)	17.73	17.91	18.24	17.74	0.180	17.99	17.80	17.81	17.99	0.128	0.395	0.331	0.064
Albumen height(mm)	7.03	6.88	7.58	7.01	0.213	7.31	6.95	6.96	7.30	0.151	0.095	0.116	0.331
Haugh unit	82.59	80.59	84.92	81.29	1.366	83.79^a^	80.96^b^	81.65	83.11	0.967	0.043	0.271	0.552
yolk index	0.0402	0.0406	0.0405	0.0401	0.000464	0.0404	0.0403	0.0404	0.0403	0.0003	0.983	0.856	0.438
yolk color	7.28	7.50	7.28	7.39	0.162	7.26	7.46	7.38	7.33	0.114	0.306	0.732	0.732
shell strength	40.82	45.05	39.18	39.18	2.660	40.33	41.71	42.86	39.18	1.891	0.430	0.163	0.430
Shell thickness	400.93	410.28	394.07	408.89	8.114	397.50	409.58	405.54	401.48	5.738	0.141	0.613	0.738
Albumen (%)	64.86	64.14	64.04	63.80	0.937	64.45	63.97	64.52	63.92	0.663	0.613	0.537	0.780
yolk (%)	26.81^a^	25.98^a^	26.90^a^	23.12^b^	0.472	26.86^a^	24.57^b^	26.40^a^	25.01^b^	0.334	<.0001	0.0044	0.0026
Shell (%)	9.77	9.88	9.06	9.78	0.235	9.42	9.83	9.82	9.42	0.166	0.085	0.092	0.203
Specific gravity	1.0858	1.0894	1.0853	1.0900	0.00167	1.086^b^	1.090^a^	1.088	1.088	0.001	0.015	1.000	0.741

CON = Control diet (without additive); EAP: *Elaeagnus angustifolia* powder; GRP: Ginger root powder; MIX = basal diet + *Elaeagnus angustifolia* powder and Ginger root powder. Means within a row with different superscripts differ significantly (*P* < 0.05).

### Blood biochemical parameters

Table 4 details the impact of the experimental diets on blood biochemical characteristics. No significant EAP × GRP interaction was detected for any blood biochemical parameter (*P* > 0.05). EAP supplementation significantly reduced serum TG concentration compared with the non-EAP groups (*P* = 0.032).

**Table 4:** Effect of dietary *Elaeagnus angustifolia* powder and ginger root powder on blood biochemical parameters and anti-oxidant status of Lohmann laying hens at 100 weeks of age.

Trait	Treatment means				SEM	Main-effect means				SEM	*P*-Values		
						EAP (g/kg of diet)		GRP (g/kg of diet)					
	CON	EAP	GRP	MIX		–	7.5	–	5		EAP	GRP	EAP × GRP
Glucose (mg/dl)	264.80	257.80	319.80	257.20	16.703	290.30	259.50	261.25	288.33	11.81	0.064	0.123	0.116
Cholesterol (mg/dl)	136.60	137.20	163.60	114.20	13.76	150.10	125.70	136.9	138.90	9.74	0.095	0.886	0.088
Triglyceride(mg/dl)	1447.20	1396.50	1578.80	1137.60	103.43	1513.00^a^	1267.05^b^	1421.85	1358.20	73.133	0.032	0.547	0.077
Calcium(mg/dl)	31.53	30.28	34.94	31.24	2.721	33.23	30.76	30.90	33.09	1.924	0.376	0.433	0.659
Phosphorous(mg/dl)	6.68	5.91	7.98	6.69	0.689	7.33	6.30	6.29	7.33	0.487	0.157	0.151	0.713
Total Protein(g/dl)	7.05	6.70	7.06	6.68	0.258	7.06	6.69	6.88	6.87	0.182	0.177	0.981	0.951
Albumin(g/dl)	2.29	2.27	2.17	2.22	0.073	2.23	2.25	2.29	2.19	0.052	0.848	0.224	0.593
AST(U/L)	139.75	145.25	97.02	158.80	18.768	118.38	152.03	142.50	127.90	13.271	0.092	0.448	0.153
ALT(U/L)	5.20	7.00	6.60	3.20	1.284	7.43	12.15	6.10	4.90	1.026	0.660	0.511	0.165
ALP (U/L)	821.40	1191.50	1029.80	1146.60	214.50	925.60	1169.10	1006.50	1088.20	151.676	0.273	0.708	0.563
ALT/AST	0.047	0.049	0.067	0.028	0.017	0.057	0.039	0.048	0.047	0.012	0.319	0.964	0.248

CON = Control diet (without additive); EAP: *Elaeagnus angustifolia* powder; GRP: Ginger root powder; MIX = basal diet + *Elaeagnus angustifolia* powder and Ginger root powder. ALT: alanine aminotransferase; AST: aspartate aminotransferase; ALP: alkaline phosphatase. Means within a row with different superscripts differ significantly (*P* < 0.05).

### Antioxidant status

Table 5 presents the effects of dietary treatments on serum antioxidant status. No significant EAP × GRP interaction was observed for any antioxidant parameter (*P* > 0.05). GRP supplementation significantly reduced serum MDA concentration (*P* = 0.003) and significantly increased SOD activity (*P* = 0.005) compared with the non-GRP groups. Similarly, EAP supplementation significantly reduced serum MDA concentration (*P* = 0.032) and significantly increased SOD activity (*P* = 0.023) compared with the non-EAP groups. TAC was not significantly affected by EAP or GRP supplementation (*P* > 0.05).

**Table 5:** The effect of adding *Elaeagnus angustifolia* powder and ginger root powder to the diet of Lohmann laying hens on serum antioxidant status (100 weeks).

Trait					SEM	Main-effect means				SEM			
	Treatment means					EAP (g/kg of diet)		GRP (g/kg of diet)			*P*-Values		
	CON	EAP	GRP	MIX		–	7.5	–	5		EAP	GRP	EAP × GRP
MDA (nmol/ml)	6.34	5.12	4.72	4.22	0.365	5.53^a^	4.67^b^	5.73^a^	4.47^b^	0.258	0.032	0.003	0.339
TAC (mmol/l)	1.21	1.29	1.54	1.51	0.151	1.373	1.402	1.25	1.53	0.107	0.850	0.087	0.701
GSH-Px (U/mL)	1203.42	1235.46	1212.72	1228.5	40.941	1206.9	1231.98	1219.44	1221.18	30.493	0.443	0.953	0.773
SOD (U/ml)	248.13	263.30	266.14	270.10	3.812	257.14^b^	266.70^a^	255.72^b^	268.12^a^	2.696	0.023	0.005	0.161

CON = Control diet (without additive); EAP: *Elaeagnus angustifolia* powder; GRP: Ginger root powder; MIX = basal diet + *Elaeagnus angustifolia* powder and Ginger root powder. MDA: Malondialdehyde; TAC: Total antioxidant capacity; GSH-Px: Glutathione peroxidase; SOD: Superoxide dismutase. Means within a row with different superscripts differ significantly (*P* < 0.05).

### Differential leukocyte counts

Table 6 presents the main and interaction effects of dietary EAP and GRP supplementation on the differential leukocyte counts of aged laying hens. No significant EAP × GRP interaction was observed for differential leukocyte counts (*P* > 0.05). Monocyte counts were significantly lower in hens receiving GRP compared with those not receiving GRP (*P* = 0.003).

**Table 6:** Effect of dietary *Elaeagnus angustifolia* powder and ginger root powder on differential Leukocyte Counts of Lohmann laying hens at 100 weeks of age.

Trait	Treatment means				SEM	Main-effect means				SEM	*P*-Values		
						EAP (g/kg of diet)		GRP (g/kg of diet)					
	CON	EAP	GRP	MIX		–	7.5	–	5		EAP	GRP	EAP × GRP
Heterophil	21.60	24.20	24.00	20.80	2.094	22.80	22.50	22.90	22.40	1.481	0.814	0.888	0.185
Lymphocyte	65.60	62.80	64.60	69.20	2.540	65.10	66.00	64.20	66.90	1.796	0.728	0.304	0.165
Monocyte	10.80	9.40	8.20	8.00	0.579	9.50	8.70	10.10^a^	8.10^b^	0.409	0.186	0.003	0.315
Eosinophil	1.40	3.00	2.60	2.60	0.667	2.00	2.80	2.20	2.60	0.471	0.248	0.557	0.248
Basophil	0.60	0.60	0.60	0.20	0.234	0.60	0.40	0.60	0.40	0.165	0.406	0.406	0.406
Heterophil/ Lymphocyte	0.330	0.384	0.382	0.314	0.0466	0.355	0.349	0.356	0.348	0.032	0.899	0.866	0.202

CON = Control diet (without additive); EAP: *Elaeagnus angustifolia* powder; GRP: Ginger root powder; MIX = basal diet + *Elaeagnus angustifolia* powder and Ginger root powder.

Means within a row with different superscripts differ significantly (*P* < 0.05).

### Ileum histomorphology parameters

Table 7 summarizes the effects of EAP and GRP on the ileal histomorphology of Lohmann LSL-Lite laying hens. The experimental diets did not significantly affect crypt depth or the villus height-to-crypt depth ratio (*P* > 0.05). A significant EAP × GRP interaction was observed for villus height, villus width, and villus surface area (*P* = 0.026, 0.010, and 0.006, respectively). Hens fed EAP or GRP alone had greater villus height and villus surface area than CON hens, whereas hens fed the MIX diet did not differ significantly from either CON or from the EAP and GRP groups. For villus width, only EAP-fed hens showed a significantly greater value than CON, while GRP and MIX did not differ significantly from CON or from EAP; the EAP main effect on villus width did not reach statistical significance (*P* = 0.055). Fig. 1 presents photomicrographs of ileal tissues across the treatment groups.

**Table 7:** Effect of dietary *Elaeagnus angustifolia* powder and ginger root powder on ileal histomorphology parameters of Lohmann laying hens at 100 weeks of age.

Trait	Treatment means				SEM	Main-effect means				SEM	*P*-Values		
						EAP (g/kg of diet)		GRP (g/kg of diet)					
	CON	EAP	GRP	MIX		–	7.5	–	5		EAP	GRP	EAP × GRP
Villus height (µm)	603.2^b^	641.59^a^	647.93^a^	627.41^ab^	11.632	625.56	634.50	662.40	637.67	8.225	0.457	0.214	0.026
Villus width (µm)	117.69^b^	123.04^a^	121.43^ab^	120.45^ab^	1.028	119.56	121.75	120.37	120.94	0.727	0.055	0.585	0.010
Crypt depth (µm)	118.56	118.94	118.94	118.72	2.409	118.75	115.83	118.75	118.83	1.704	0.976	0.973	0.904
Villus height/ Crypt depth	5.09	5.40	5.45	5.30	0.132	5.27	5.35	5.25	5.37	0.094	0.563	0.359	0.113
Villus Surface area (mm^2^)	0.223^b^	0.248^a^	0.247^a^	0.237^ab^	0.0052	0.235	0.243	0.236	0.242	0.0037	0.167	0.226	0.006

CON = Control diet (without additive); EAP: *Elaeagnus angustifolia* powder; GRP: Ginger root powder; MIX = basal diet + *Elaeagnus angustifolia* powder and Ginger root powder.

*n* = 6 replicates/treatment (one bird per replicate cage), and that each value represents the mean of 10 villus-crypt units measured per bird, rather than individual villus measurements.

Means within a row with different superscripts differ significantly (*P* < 0.05).

**Fig. 1:**
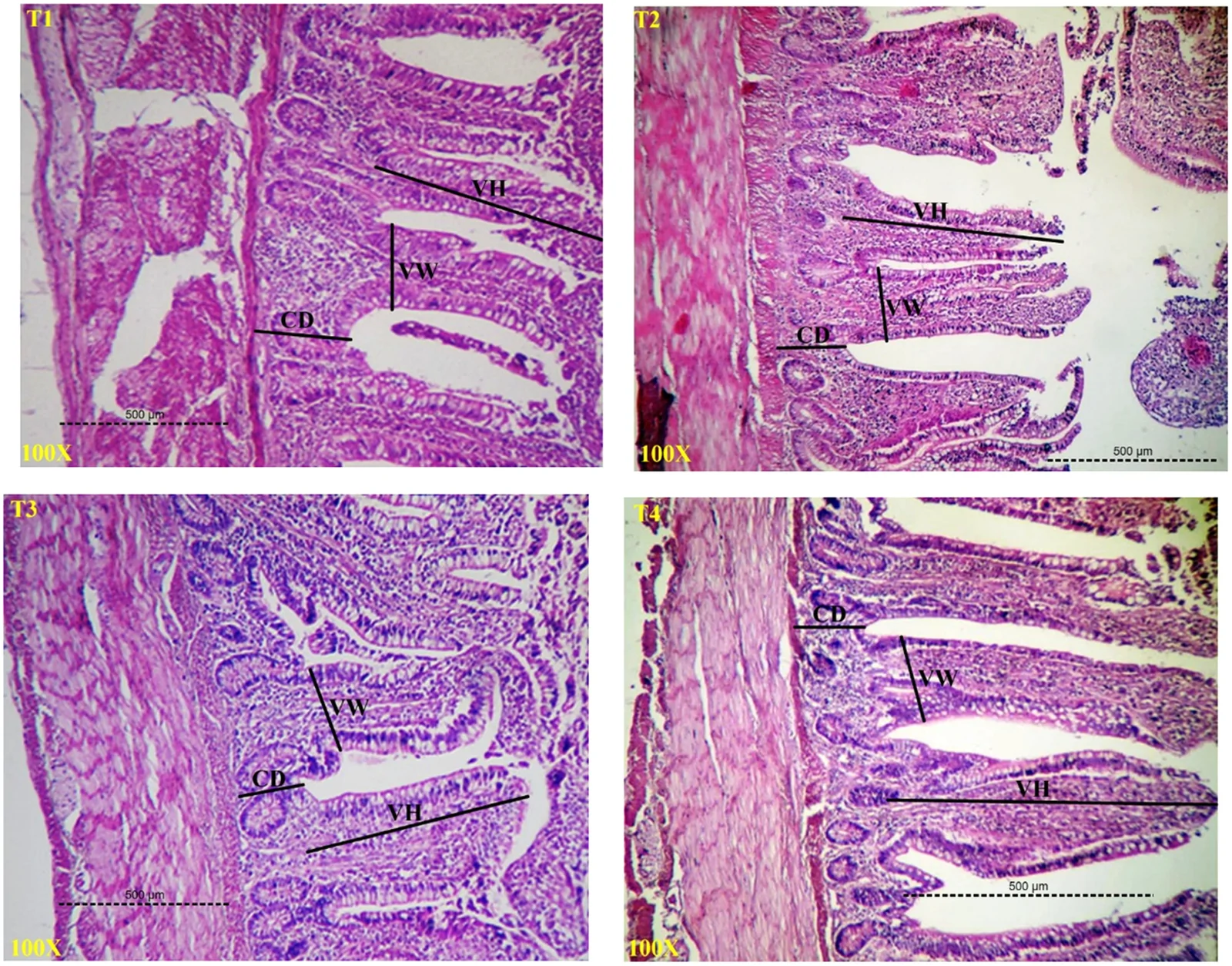
photomicrographs of ileal tissues across various treatment: T1 (control), T2 (*Elaeagnus angustifolia* powder), T3 (Ginger root powder), and T4 (*Elaeagnus angustifolia* powder + Ginger root powder). VH: Villus height, CD: Crypt depth, and VE: Villus width.

### Organ characteristics

Table 8 presents the main and interaction effects of dietary supplementation with EAP and GRP on organ characteristics of Lohmann LSL-Lite laying hens. A significant EAP × GRP interaction was observed for pancreas weight (*P* = 0.002); hens fed the MIX diet had a higher pancreas weight than hens fed GRP alone, while CON and EAP-fed hens did not differ significantly from either group. EAP supplementation significantly increased cecum weight, from 14.65% (without EAP) to 21.98% (with EAP) (*P* = 0.033).

**Table 8:** Effect of dietary *Elaeagnus angustifolia* powder and ginger root powder on organ characteristics of Lohmann laying hens at 100 weeks of age.

Trait	Treatment means				SEM	Main-effect means				SEM	*P*-Values		
						EAP (g/kg of diet)		GRP (g/kg of diet)					
	CON	EAP	GRP	MIX		–	7.5	–	5		EAP	GRP	EAP × GRP
Carcass (%)	61.99	65.99	64.05	58.78	3.562	63.02	62.38	63.98	61.41	2.519	0.862	0.483	0.218
Gizzard (%)	1.46	1.60	1.36	1.69	0.117	1.41	1.65	1.532	1.528	0.083	0.067	0.967	0.430
Pancreases (%)	0.289^ab^	0.280^ab^	0.195b	0.365^a^	0.0825	0.242^b^	0.323^a^	0.284	0.280	0.016	0.004	0.846	0.002
Liver (%)	2.23	2.48	2.11	2.68	0.319	2.17	2.58	2.35	2.39	0.226	0.224	0.901	0.620
Heart (%)	0.396	0.466	0.443	0.384	0.031	0.419	0.425	0.43	0.41	0.022	0.851	0.575	0.059
Duodenum (%)	20.97	18.13	32.07	17.74	5.163	26.52	17.94	19.55	24.90	3.652	0.122	0.320	0.289
Jejunum (%)	34.49	34.16	33.10	32.14	1.503	33.79	33.15	34.32	32.62	1.063	0.678	0.279	0.839
Ileum (%)	30.60	25.02	29.16	28.84	2.411	29.87	26.93	27.81	28.99	1.704	0.245	0.632	0.297
Ceca (%)	13.93	22.69	15.36	21.27	3.046	14.65^b^	21.98^a^	18.31	18.32	2.153	0.033	0.999	0.649

CON = Control diet (without additive); EAP: *Elaeagnus angustifolia* powder; GRP: Ginger root powder; MIX = basal diet + *Elaeagnus angustifolia* powder and Ginger root powder*.*

Carcass, gizzard, pancreas, liver, and heart weights are expressed as a percentage of live body weight. Duodenum, jejunum, ileum, and cecum weights are expressed as a percentage of total intestinal weight. Means within a row with different superscripts differ significantly (*P* < 0.05).

## Discussion

Maintaining high laying performance and eggshell quality in aging hens remains a major challenge in commercial poultry production. With advancing age, hens commonly exhibit declines in egg production, shell strength, and shell thickness (Nik et al., 2025). In the present study, supplementation with EAP improved egg production and FCR during the second period (97–100 wk), suggesting enhanced productive efficiency in older birds. As the proximate analysis indicated that EAP contained 6.64% crude fiber, one possible mechanism is that structural fiber components stimulated mechanical activity in the foregut, particularly the gizzard and crop, thereby promoting digestive enzyme secretion and microbial fermentation in the hindgut (Kheravii et al., 2018). Another possible mechanism may be due to the improved height, width, and surface area of ​​the villi in this study. Furthermore, Paiva et al. (2014) indicate that elongated villi and shallower crypts are indicative of more mature and developed intestinal mucosal cells, thereby suggesting a heightened capacity for intestinal digestion and absorption. Additionally, bioactive compounds present in EAP, including flavonoids, phenolics, sterols, and terpenoids (Afzali et al., 2016), may have further supported metabolic efficiency and gut health. Although Afzali et al. (2016) reported similar improvements in performance, Basiriyan et al. (2021) found no effect of EAP in broilers, which may reflect differences in species, age, or physiological status.

Eggshell quality also responded to EAP supplementation, with specific gravity increasing significantly from 1.086 in CON to 1.090 in the EAP-treated group (*P* = 0.015). As specific gravity reflects the proportion of shell relative to total egg components (Butcher and Miles, 1991), this finding may reflect a subtle improvement in shell quality with EAP supplementation.

A significant EAP × GRP interaction was also detected for yolk percentage, with the MIX group exhibiting a lower value than the other treatment groups. This reduction may be associated with the concurrent decrease in serum TG observed with EAP supplementation, potentially reflecting altered lipid metabolism or excretion (Afzali et al., 2016); as lipid excretion was not directly assessed, this interpretation remains hypothesis-generating and warrants further investigation. The combined use of EAP and GRP did not produce strong synergistic effects for most egg quality traits, suggesting overlapping mechanisms of action.

FI and feed efficiency are critical economic parameters, accounting for up to 70% of production costs (Spring, 2013). In this study, FCR improved significantly with GRP supplementation over the entire experimental period (92–100 wk; *P* = 0.046), indicating enhanced feed efficiency. Ginger characteristic pungent flavor and aroma are primarily attributed to gingerols and shogaols (Ajanaku et al., 2022; Aleem et al., 2020), which have been reported to affect feed palatability in poultry in some studies (Niknafs et al., 2023).

In this study, improvements in egg production and FCR were observed in birds fed the experimental diets. Ginger rhizome has been reported to improve nutrient uptake efficiency by slowing the transit of feed within the gastrointestinal tract (Vyas et al., 2018), which may consequently lower FCR and enhance overall performance.

Ginger has been previously reported to support in digestion by stimulating the secretion of digestive juices and increasing the activity of enzymes such as lipase and maltase. Its active compounds may also interact with serotonin receptors, improving gut function and promoting growth (Ibtisham et al., 2019). This increased enzymatic activity has been shown to support nutrient absorption and lead to increased egg production (Incharoen and Yamauchi, 2009). In addition, ginger is able to increase saliva production, which may further enhance the activity of digestive enzymes and overall digestive efficiency (Siregar et al., 2024). Studies have shown that herbal feed additives, including ginger, improve nutrient digestion, growth rate and FCR (Yadav et al., 2016). These findings are consistent with our results regarding the reduction in FCR under the influence of GRP.

In the current experiment, villus height, width, and villus surface area increased significantly under the influence of experimental treatment compared to the CON. Gingerol has been reported to exhibit antimicrobial activity by disrupting bacterial membranes, inhibiting enzyme systems, and interfering with quorum sensing (Dosu et al., 2023; Elfaky et al., 2024). Furthermore, ginger has been reported in previous studies to support gut health by reducing pathogen load (Kairalla et al., 2022; Vyas et al., 2018). Together, these actions improve gut health and the bird’s ability to utilize nutrients. As gut microbiota composition was not evaluated in the present study, any contribution of these antimicrobial mechanisms to the observed results remains hypothetical.

The antioxidant effects of EAP and GRP also played a significant role in improving physiological status. Birds receiving these supplements showed reduced MDA levels and increased antioxidant marker such as SOD. Reduced MDA indicates lower lipid peroxidation and improved cellular integrity (Yang et al., 2010). Among the bioactive constituents of ginger, 6-shogaol has been reported to possess the strongest antioxidant and anti-inflammatory properties, attributed to its α, β-unsaturated ketone moiety (Dugasani et al., 2010). Both 6-gingerol and 6-shogaol have been reported to protect against DNA damage and lipid peroxidation (Lu et al., 2014). These previously reported mechanisms may partly explain the antioxidant effects of GRP observed in the present study.

Wang et al. (2013) reported that EAP fruit and seed exhibit high antioxidant activity. Previous studies have similarly reported that EAP fruit contains high concentrations of antioxidant, phenolic, and flavonoid compounds (Cansev et al., 2011; Hamidpour et al., 2017; Wang et al., 2013). Emami et al. (2024) reported that EAP has antioxidant, antibacterial, and anticancer properties.

Gingerol, gingerdiol, gingerdione, terpenes, oleoresin, and shogaol in ginger can increase the activity of digestive enzymes (Siregar et al., 2024) and exhibit antibacterial (Beristain-Bauza et al., 2019), immunostimulatory (Si et al., 2018), and antiparasitic (Fu et al., 2019) properties. As a result, these components contribute to improved growth performance, immunity, and antioxidant capacity in animal subjects (Ogbuewu et al., 2020).

The increase in pancreatic weight observed in birds receiving EAP and GRP may hypothetically reflect enhanced digestive enzyme production, contributing to improved feed utilization. Moreover, both additives appear to reduce oxidative stress and inflammation, which are known to divert energy away from productive processes (Surai et al., 2019).

Moreover, monocyte counts were reduced in birds receiving GRP, suggesting a potential immunomodulatory effect. Since elevated monocyte levels are often associated with inflammation and stress (Rana et al., 2018), their reduction may indicate improved physiological homeostasis. This interpretation is supported by the observed improvements antioxidant status.

## Conclusions

Dietary EAP (7.5 g/kg) and/or GRP (5.0 g/kg) improved aged laying hens' performance, egg quality, antioxidant biomarkers, ileal villus morphology, and some organ characteristics. However, not all measured variables were significantly affected. The observed improvements in performance may be partly associated with altered antioxidant status and gut morphology, although direct evidence for changes in digestive enzyme activity, nutrient digestibility, intestinal permeability, microbiota, or molecular antioxidant pathways was not obtained in this study. Further research is needed to elucidate underlying molecular mechanisms, including antioxidant signaling, gut microbiota, enzyme activity, and hepatic lipid metabolism. These findings are specific to the doses, botanical preparations, and aged Lohmann LSL-Lite hens used in this study.

## Author contributions

**Zahra Hamzehee:** Analysis of data, Writing the manuscript draft, Reviewing and explicitly approving the exact, final version of the manuscript before it goes to press. Agreeing to be personally and publicly responsible for all aspects of the work, ensuring that any questions regarding the accuracy or integrity of the research are thoroughly investigated and resolved.

**Mehran Torki:** Substantive input into the conception or design of the work, interpretation of data. Reviewing the manuscript draft critically for crucial intellectual content. Reviewing and explicitly approving the exact, final version of the manuscript before it goes to press. Agreeing to be personally and publicly responsible for all aspects of the work, ensuring that any questions regarding the accuracy or integrity of the research are thoroughly investigated and resolved.

**Khodabakhsh Rashidi:** Substantive input into the interpretation of data. Reviewing the manuscript draft critically for crucial intellectual content. Reviewing and explicitly approving the exact, final version of the manuscript before it goes to press. Agreeing to be personally and publicly responsible for all aspects of the work, ensuring that any questions regarding the accuracy or integrity of the research are thoroughly investigated and resolved.

**Hamid Pajavand:** Substantive input into the interpretation of data. Reviewing the manuscript draft critically for crucial intellectual content. Reviewing and explicitly approving the exact, final version of the manuscript before it goes to press. Agreeing to be personally and publicly responsible for all aspects of the work, ensuring that any questions regarding the accuracy or integrity of the research are thoroughly investigated and resolved.

## Declaration of generative AI and AI-assisted technologies in the writing process

During the preparation of this work the author(s) used Sider in order to paraphrase sentences and improve grammar and language clarity. After using this tool, the author(s) reviewed and edited the content as needed and take(s) full responsibility for the content of the published article.

## Disclosures

The authors declare that they have no known competing financial interests or personal relationships that could have appeared to influence the work reported in this paper.
